# Contributions of Trustworthiness, Health Literacy, and Self-Efficacy in Communicating With COVID-19 Vaccine–Hesitant Audiences: Web-Based Survey Study

**DOI:** 10.2196/38076

**Published:** 2022-08-01

**Authors:** Sitara M Weerakoon, Mike Henson-Garcia, Melissa A Valerio-Shewmaker, Sarah E Messiah, Gregory Knell

**Affiliations:** 1 Center for Pediatric Population Health School of Public Health and Children's Health System of Texas University of Texas Health Science Center, Dallas Campus Dallas, TX United States; 2 Department of Health Promotion and Behavioral Science School of Public Health University of Texas Health Science Center, Brownsville Campus Brownsville, TX United States; 3 Children’s Health Andrews Institute for Orthopaedics and Sports Medicine Plano, TX United States

**Keywords:** vaccine hesitancy, health literacy, COVID-19, COVID-19 vaccine hesitancy, health communication, vaccination, health professional, health information

## Abstract

**Background:**

Large-scale health communication challenges during the COVID-19 pandemic, such as widespread misinformation and distrust in health care professionals, have influenced reluctance to take the COVID-19 vaccine, also known as vaccine hesitancy. Trust in health professionals, adequate health literacy, and high self-efficacy are key components of actively pursuing preventative and protective health care measures. These factors may be associated with intentions to seek and complete a COVID-19 vaccine dosing.

**Objective:**

The objective of this analysis was to identify factors associated with COVID-19 vaccine hesitancy.

**Methods:**

In February 2021, US adults (N=5872) responded to a web-based survey on COVID-19 vaccine hesitancy and components of health communication (trust in sources of health information, health literacy, and self-efficacy). Multivariable logistic regression models were used to explore associations between these factors and vaccine hesitancy while adjusting for key demographics. We hypothesized that low levels of trust, health literacy, and self-efficacy would be associated with increased vaccine hesitancy.

**Results:**

The adjusted odds of vaccine hesitancy was greater among those who placed little to no trust in health professionals compared to those who held a lot of trust (adjusted odds ratio [AOR] 8.54, 95% CI 6.52-11.19). The odds of vaccine hesitancy was also greater among those who felt frustrated about finding health information compared to those who did not (AOR 2.10, 95% CI 1.62-2.70). Participants who had little to no confidence in receiving health advice or information had greater odds of vaccine hesitancy compared to those who had a lot of confidence (AOR 3.05, 95% CI 2.34-3.97).

**Conclusions:**

This study underscores the importance of trust between health professionals and their patients, and a need for improving health literacy regarding vaccines. Perceptions of mistrust and low levels of health literacy were associated with high levels of vaccine hesitancy, providing empirical support of framing these factors as perceived barriers to vaccine uptake.

## Introduction

### Background

Between March 2020 and February 2022, the spread of the SARS-CoV-2 virus led to over 500 million cases of COVID-19 disease and over 6 million deaths globally [1]. Since the start of the pandemic, efforts to slow or prevent the spread of COVID-19 have included business lockdowns and stay-at-home policies; international border closings and movement restrictions and mandating or encouraging mask wearing, frequent handwashing, and physical distancing imposed by federal, state, and local governments [2]. More recently, COVID-19 vaccine acceptance and uptake as well as booster completion have become critical components to decreasing the incidence of COVID-19 and ultimately ending the pandemic. However, within the United States, there have been large-scale health communication challenges regarding the COVID-19 vaccines that have impeded efforts toward achieving herd immunity. These challenges include concerns regarding vaccine safety, the spread of misinformation, belief in personal freedom and choice, and lack of access to reliable public health information [3,4]. A reluctance to taking a vaccine, also known as vaccine hesitancy, poses significant dangers to public safety during a pandemic given that unvaccinated individuals can more easily contract and spread disease to others and may contribute to development of strains not contained by the vaccine [5].

### Vaccination Rates and Related Factors

In the United States, vaccine rollout began in late December 2019 prioritizing health care professionals and residents of long-term care facilities before expanding to other essential workers and businesses, and eventually to all adults. COVID-19 vaccination rates have consistently climbed since the vaccine was made available to all persons over the age of 18 years on April 19, 2021 [6]; as of February 2022, over 550 million doses of the vaccine have been administered, amounting to approximately 78% of the population with at least one dose. From April 2021 to the present, there have been challenges in reaching those who might be vaccine-hesitant. Most commonly reported reasons for vaccine hesitancy include skepticism of the vaccine, more specifically a lack of knowledge surrounding the components and safety of the vaccine [5,7,8]. Further, some are now using the term “pandemic of misinformation” to describe the circulation of mixed messaging and potentially inaccurate news being shared via social media [9-11]. The Edelman Trust Barometer Report, one of the longest-running surveys on trust, found that globally in 2021, over 70% of respondents were worried about the spread of false news, and reported high levels of distrust in government entities and media [12]. This skepticism coupled with the spread of misinformation have led to widespread reluctance in uptake of the COVID-19 vaccine across the United States.

### Definitions

#### Source Credibility and Health Literacy

Health communication refers to the study and practice of delivering information intended to promote health and well-being among a particular audience. Activities in the health communication space range from large-scale, multifaceted public health campaigns to private patient-provider interactions [13]. Broadly speaking, health communication seeks to improve public health through increasing awareness of a health issue or influencing individual behaviors and practices through a variety of messaging approaches (eg, demonstrating the benefits of a health intervention). Appraisal of health messages by a particular audience depends on many different intersecting factors, including source credibility and health literacy [4,13-15].

Source credibility—or the extent to which an information source is perceived as believable [16]—is one factor that is thought to influence message acceptance by an audience [17]. Theoretically, the vast majority of conceptualizations surrounding source credibility distinguish between two overlapping yet distinct perceptions involved in gauging credibility: (1) trustworthiness of the source and (2) expertise of the source. Within the credibility literature, trustworthiness has been defined as “the degree of confidence in the communicator’s intent to communicate the assertions he considers most valid,” whereas expertise is understood to mean “the extent to which a communicator is perceived to be a source of valid assertions” [18]. Moreover, while information accuracy is thought to be involved in the message appraisal process, the degree to which information is accurate is conceptually different from perceptions around the credibility of a source [16], which has important implications for understanding and responding to disinformation and misinformation campaigns related to COVID-19 vaccinations.

In the context of COVID-19 vaccine hesitancy, some preliminary qualitative findings from Bateman and colleagues [19] suggested that mistrust is an important factor that may be influencing the widespread COVID-19 vaccine hesitancy among Latinx and African American communities in the Deep South. More specifically, the authors identified interesting subthemes within the data differentiating between (1) historical *mis*trust, or *mis*trust that is related to systemic racism and historical oppression; (2) vaccine development *mis*trust; and (3) *mis*trust in politicians [19]. However, another survey among a US sample living in Phoenix, Arizona, and New York City, New York, found that those who held higher trust in government were less likely to intend to get vaccinated against COVID-19 [20]. Several studies have shown that, in multiple contexts, trust in self-seeking information is critical to behavior change [21-23]. Therefore, given these preliminary findings, there exists a critical need to clarify the way source trustworthiness is associated with COVID-19 vaccine hesitancy within the United States.

Health literacy is also a critical factor that can either facilitate or thwart the process of appraising health information. Aligning with the findings from a systematic review [24] examining common definitions and conceptualizations related to the construct, we view health literacy as being

linked to literacy and entails people’s knowledge, motivation and competences to access, understand, appraise, and apply health information in order to make judgments and take decisions in everyday life concerning healthcare, disease prevention and health promotion to maintain or improve quality of life during the life course.

Some recent evidence demonstrates robust and intricate relationships between health literacy and COVID-19 vaccine hesitancy. For instance, Kricorian and colleagues [25] determined that vaccine-hesitant individuals were more likely to find information related to the COVID-19 vaccine difficult to comprehend compared to individuals who did not express hesitancy. Furthermore, in a study of adults residing in China, researchers determined that higher levels of health literacy were protective against COVID-19 vaccine-hesitant attitudes, although this protective effect was only observed among individuals with low to moderate levels of stress [26]. Additionally, Turhan and colleagues [27] determined that a sample of Turkish social media users were more likely to report hesitant attitudes if they held low levels of health literacy and high levels of distrust in government. Their study further showed that “health literacy mediated the relationship between health care system distrust and vaccine hesitancy,” which implicates health literacy as an important factor to be considered in the design of health communication campaigns and health promotion interventions that attempt to modify COVID-19 vaccine hesitancy among communities [27].

#### Self-efficacy Theory

Self-efficacy is a cornerstone construct in the study of motivation and behavior. Originally stemming from Albert Bandura’s Social Cognitive Theory developed in the 1960s, self-efficacy refers to an individual’s perceptions of their capacity and capability to learn or behave in a particular way [28]. Since its inception, the construct has been applied and adapted to various theories of health behavior, including the Health Belief Model [29], Theory of Planned Behavior [30], and Transtheoretical Model of Behavior Change [31].

High levels of self-efficacy have been previously associated with intentions to be vaccinated against COVID-19. For example, Guidry et al [32] determined that those who scored high in self-efficacy to overcome vaccination barriers were more likely to form intentions about receiving the COVID-19 vaccine. Another online study of Korean internet users examined the relationships between self-efficacy and health literacy in the context of COVID-19 vaccine hesitancy, and reported that self-efficacy was negatively associated with hesitancy. These authors also reported that individuals who maintained high levels of eHealth literacy—a specific subtype of health literacy—were more likely to exhibit high levels of self-efficacy. Aligning with evidence from other health contexts, these scholars ultimately advance that given their findings, it is important for interventionists to target health literacy in prevention activities as a potential mechanism to enhance an individual’s self-efficacy and thus prevent COVID-19 vaccine hesitancy [33]. However, other studies—such as that conducted by McElfish et al [34] with a sample of individuals residing in Arkansas, United States—have found no significant difference in COVID-19 vaccine hesitancy according to levels of protection self-efficacy, operationalized as an individual’s belief in their ability to protect themselves against COVID-19. Given this mixed evidence, it is imperative to further elucidate the role self-efficacy has in the larger issue of COVID-19 vaccine hesitancy.

### Purpose and Hypothesis

Trust in sources of health information, adequate health literacy, and high self-efficacy are key components of actively pursuing preventative and protective health care measures. These factors may be associated with intentions to seek and complete a COVID-19 vaccine dosing. In this study, we (1) evaluated the relative levels of these constructs among a population of US adults who use social media and (2) quantified the relationships between these constructs on COVID-19 vaccine hesitancy. We hypothesized that distrust in sources of health information, inadequate health literacy, and low self-efficacy are associated with being unsure about or choosing not to get the COVID-19 vaccine.

## Methods

### Sample Description

From February 3 to March 2, 2021, a web-based self-report survey was disseminated through social media platforms (eg, Facebook, Instagram, LinkedIn, Twitter) and through email lists accessible to the authors. Specifically, the survey was disseminated on the lead author and principal investigator’s personal and professional social media accounts. Social media users could follow the shared link and were first asked eligibility questions regarding their age and US residence status. If users were younger than 18 years or resided outside of the United States, they could not proceed with the survey. No incentives were provided for taking the survey.

### Ethical Considerations

Institutional review board approval was obtained prior to survey dissemination (HSC-SPH-20-0346). Participants met eligibility criteria if they were 18 years or older and resided within the United States. Before the start of the survey, participants were informed about the purpose and estimated time required to complete survey, that their participation was voluntary, and that they could skip any questions they did not wish to answer. All respondents provided written consent to participate before proceeding to the questions.

### Measures

#### Primary Outcome

The survey asked participants “Have you taken the COVID-19 vaccine?” Participants could select “Yes” or “No”; if a participant selected “No,” they were then prompted with the question “Will you receive the COVID-19 vaccine when it is available to you?”; participants could then select “Yes,” “No,” “Unsure,” or “I prefer not to say.” Vaccine hesitancy is understood to mean reluctance to take a vaccine; therefore, those who selected “No” or “Unsure” about their plans to receive the vaccine when available were considered to be vaccine-hesitant. Those who either already received or planned to receive the vaccine were the referent group. The “I prefer not to say” responses were treated as missing since the attitude toward the vaccine could not be determined (n=7; 0.3%).

#### Exposures

There were several measurements of the two major components of health communication that were explored in this analysis. All health communication questions were adapted from the Health Information National Trends Survey questionnaire [35]. The survey asked participants to report on a Likert scale (ie, *A lot*, *Some*, *A little*, and *Not at all*) their level of trust in the following sources of health or medical information: (1) government agencies, (2) a doctor or health professional, (3) family or friends, and (4) religious organizations and leaders. Responses *Some*, *A little*, and *Not at all* were collapsed to represent “Not a lot of trust or not at all”; the variable was a binary variable to reflect “A lot of trust” and “Not a lot or not at all.” Participants were also asked about health communication accessibility and comprehension as a measurement of health literacy. Specifically, the survey asked participants to report on a Likert scale (ie, *Strongly agree*, *Somewhat agree*, *Somewhat disagree*, and *Strongly disagree*) how much they agreed or disagreed with the following statements based on the results of their most recent search for information about health or medical topics: (1) *It takes a lot of effort to get health or medical information you need* and (2) *Felt frustrated during your search for health or medical information*. Responses *Strongly agree* and *Somewhat agree* were collapsed to represent “Agree,” while the responses *Somewhat disagree* and *Strongly disagree* were collapsed to represent “Disagree”; the variable was a binary variable to reflect “Agree” and “Disagree.” Finally, the survey asked participants a question regarding confidence in one’s ability to find, understand, and make health-related choices (ie, self-efficacy). The survey asked participants to report on a Likert scale (ie, *Strongly agree*, *Somewhat agree*, *Somewhat disagree*, and *Strongly disagree*) how much they agreed or disagreed with the following statement: *Confidence in getting advice or information about health or medical topics*. Responses *Strongly agree* and *Somewhat agree* were collapsed to represent “Agree,” while the responses *Somewhat disagree* and *Strongly disagree* were collapsed to represent “Disagree”; the variable was a binary variable to reflect “Agree” and “Disagree.”

Although these Likert-scale questions have previously been analyzed both on a continuous scale and categorically [36,37], the response categories for the exposure variables were collapsed due to one or more of the following reasons: the sample sizes were small (<1%) within categories, the distinction between “strongly agree” and “agree” (and other responses) was minor or indistinguishable, or analyses were run that included the original exposure variable categories and odds of vaccine hesitancy did not vary significantly across the categories that were ultimately collapsed (results not shown).

#### Covariates

Sociodemographic variables (ie, covariates) included age in years (on a continuous scale), self-identified sex (male/female), race/ethnicity (white non-Hispanic/other), education level (less than college/college or more), and annual household income based on 2019 Medicaid expansion guidelines (138% below poverty; <US $30,000/year; US $30,000-$80,000/year; and >US $80,000) [38].

### Statistical Analyses

Summary statistics are used to describe the study sample. Frequencies were tabulated and *χ*^2^ tests were performed for each health communication and literacy factor and the sociodemographics and outcome (vaccine hesitancy). Multivariable logistic regression was used to further explore the associations between the outcome and each exposure. Separate models were run for each exposure while adjusting for key demographics (age, sex, race, income, and education level). A posthoc exploratory analysis was performed to find political ideology and gender differences in vaccine-hesitant status (results not shown). Analyses were performed using Stata statistical software version 15.1.

## Results

The survey instrument captured a total of 6471 respondents; of those, 6452 (99.71%) met the eligibility criteria and 5872 (90.74%) consented to participate. Ultimately, 5356 participants responded to the series of vaccine plans–related questions, which was the final analytical sample size. The mean age of the sample was 45 years (SD 11.5); the majority were female and non-Hispanic white. As of February 2021, just over 50% of the sample had already received at least one dose of the COVID-19 vaccine. Of those who did not, approximately 5% reported that they were not going to receive it or were unsure (vaccine-hesitant). Over 50% of the total sample placed little to no trust in government agencies for health or medical information and 79% placed a lot of trust in doctors or health professionals (Table 1).

**Table 1:** Descriptive characteristics of the study sample (N=5872).

Characteristics				Value
**Sociodemographics**				
	Age (years), mean (SD)			44.8 (11.5)
	**Sex, n (%)**			
		Male	665 (12.2)	
		Female	4782 (87.8)	
	**Race/ethnicity, n (%)**			
		Non-Hispanic white	5107 (93.9)	
		Other	332 (6.1)	
	**Educational attainment, n (%)**			
		Less than college	675 (12.5)	
		College or more	4733 (87.5)	
	**Annual household income prior to the pandemic (US $), n (%)**			
		<30,000	131 (2.5)	
		30,000-$80,000	987 (18.6)	
		>$80,000	4190 (78.9)	
	**Already received the COVID-19 vaccine, n (%)**			
		Yes	2744 (50.7)	
		No	2665 (49.3)	
	**Plan to get a COVID-19 vaccine when available as of February 2021, n (%)**			
		Yes, or already received	5077 (94.8)	
		No or unsure (vaccine-hesitant)	279 (5.2)	
**Trust in resources regarding health or medical information, n (%)**				
	**Government agencies**			
		A lot	2136 (36.4)	
		Not a lot or not at all	3075 (52.4)	
	**A doctor or other health professional**			
		A lot	4612 (78.5)	
		Not a lot or not at all	604 (10.3)	
	**Family or friends**			
		A lot	176 (3.0)	
		Not a lot or not at all	5035 (85.8)	
	**Religious organizations and leaders**			
		A lot	94 (1.6)	
		Not a lot or not at all	5102 (86.9)	
**Health literacy, n (%)**				
	**It takes a lot of effort to get health or medical information you need**			
		Disagree	3354 (57.1)	
		Agree	1834 (31.2)	
	**Felt frustrated during your search for health or medical information**			
		Disagree	3490 (59.4)	
		Agree	1664 (28.3)	
	**Confidence in getting advice or information about health or medical topics**			
		Extremely or very confident	4193 (71.4)	
		Little to no confidence	992 (16.9)	

Overall, the vaccine-hesitant sample appeared to be younger, disproportionality more male, disproportionately having less than a college degree, and disproportionately having an annual household income of US $30,000-$80,000 than the nonvaccine-hesitant sample. Lack of trust in doctors and health professionals was more prevalent in the vaccine-hesitant compared to those who already received a COVID-19 vaccine or had plans to. More vaccine-hesitant participants reported that it takes a lot of effort to access necessary health information and felt frustrated during their search for health information compared to those who already received or planned to receive the vaccine. Over three-quarters of those who already received or planned to receive the vaccine were extremely or very confident in getting advice or information about health or medical topics compared to only slightly more than half of those who were vaccine-hesitant (Table 2).

After adjusting for covariates, the odds of being vaccine-hesitant were greater among those who lacked trust in doctors or health professionals and those who felt frustrated about finding health information, as compared to those who placed a lot of trust in either resource. Similarly, the adjusted odds of being vaccine-hesitant were almost eight times greater among those who placed little to no trust in government agencies regarding health or medical information compared to those who placed a lot of trust in government agencies. The adjusted odds of being vaccine-hesitant were 75% lower among those who placed little to no trust in religious leaders and organizations, compared to those who placed a lot of trust in religious leaders and organizations. Participants who had little to no confidence in getting advice or information on health topics had 3.05 greater adjusted odds of vaccine hesitancy compared to those who already received or planned to receive the vaccine. Participants who felt frustrated during their search for health or medical information had 2.10 greater odds of being vaccine-hesitant, and participants who agreed that it takes a lot of effort to obtain the health or medical information they need had 1.59 times greater odds of being-vaccine hesitant compared to the referent group after adjusting for covariates (Table 3).

**Table 2:** Bivariate relationships between planning to get the COVID-19 vaccine when available as of February 2021 and health communication factors (N=5356).

Characteristics			Planning to get COVID-19 vaccine		*P* value
			Yes, or already received (n=5077)	Vaccine-hesitant (no or unsure) (n=279)	
**Sociodemographics**					
	Age (years), mean (SD)		44.8 (11.6)	42.6 (9.8)	.002
	**Sex, n (%)**				<.001
		Male	597 (11.8)	55 (19.7)	
		Female	4477 (88.2)	224 (80.3)	
	**Race/ethnicity, n (%)**				.81
		Non-Hispanic white	4757 (93.9)	260 (93.5)	
		Other	310 (6.1)	18 (6.5)	
	**Educational attainment, n(%)**				<.001
		Less than college	601 (11.9)	62 (22.3)	
		College or more	4467 (88.1)	216 (77.7)	
	**Annual household income prior to the pandemic (US $), n (%)**				.01
		<30,000	122 (2.5)	7 (2.6)	
		30,000-80,000	908 (18.2)	69 (25.3)	
		>80,000	3947 (79.3)	197 (72.2)	
**Trust in these resources regarding health or medical information, n (%)**					
	**Government agencies**				<.001
		A lot	2084 (41.1)	23 (8.2)	
		Not a lot or not at all	2787 (54.9)	242 (86.7)	
	**A doctor or other health professional, n (%)**				<.001
		A lot	4408 (86.8)	139 (49.8)	
		Not a lot or not at all	467 (9.2)	126 (45.2)	
	**Family or friends**				.49
		A lot	166 (3.3)	7 (2.5)	
		Not a lot or not at all	4703 (92.6)	259 (92.8)	
	**Religious organizations and leaders**				<.001
		A lot	75 (1.5)	17 (6.1)	
		Not a lot or not at all	4781 (94.2)	249 (89.3)	
**Health literacy, n (%)**					
	**It takes a lot of effort to get health or medical information you need**				<.001
		Disagree	3174 (62.5)	142 (50.1)	
		Agree	1676 (33.0)	123 (44.1)	
	**Felt frustrated during your search for health or medical information**				<.001
		Disagree	3310 (65.2)	133 (47.7)	
		Agree	1508 (29.7)	131 (47.0)	
	**Confidence in getting advice or information about health or medical topics**				<.001
		Extremely or very confident	3988 (78.6)	152 (54.5)	
		Little to no confidence	860 (17.0)	110 (39.4)	

**Table 3:** Multivariable odds of COVID-19 vaccination hesitancy when available as of February 2021 (N=5356).

Variable^a^				Vaccine-hesitant^b^, OR^c^ (95% CI)
**Trust in these resources regarding health or medical information**				
	**Government agencies**			
		A lot	Reference	
		Not a lot or not at all	7.79 (5.05-12.02)	
	**A doctor or other health professional**			
		A lot	Reference	
		Not a lot or not at all	8.54 (6.52-11.19)	
	**Family or friends**			
		A lot	Reference	
		Not a lot or not at all	1.30 (0.60-2.83)	
	**Religious organizations and leaders**			
		A lot	Reference	
		Not a lot or not at all	0.25 (0.14-0.44)	
**Health literacy**				
	**It takes a lot of effort to get health or medical information you need**			
		Disagree	Reference	
		Agree	1.59 (1.23-2.04)	
	**Felt frustrated during your search for health or medical information**			
		Disagree	Reference	
		Agree	2.10 (1.62-2.70)	
	**Confidence in getting advice or information about health or medical topics**			
		Extremely or very confident	Reference	
		Little to no confidence	3.05 (2.34-3.97)	

^a^Separate models were run for each variable and adjusted for age, sex, race, income, and education level.

^b^Vaccine-hesitant: those who reported “no” or “unsure” regarding plans to get the COVID-19 vaccination.

^c^OR: odds ratio; odds are in relation to those who already received or planned to receive the vaccine.

## Discussion

### Principal Findings

This analysis explored components of health communication that are related to vaccine hesitancy among a large convenience sample of US adults. Distrust in health professionals, lack of access to health information, and inadequate health literacy were significantly associated with vaccine hesitancy. Specifically, trust in health care professionals is a fundamental piece of the patient-doctor relationship that can significantly impact personal and public health [39]. According to a 2018 New York Times article, trust in health professionals is on the decline in the United States [40]. According to a 1966 poll, 73% of respondents reported confidence in medical professionals, and by 2012 that number had dropped to 34% [41]. Studies and surveys have shown that trust in the health care system is directly related to following treatment plans, consistently taking medications, and health competence [42,43]. Specifically in the context of COVID-19, Antinyan et al [44] found that, on a global scale, having a health care system that citizens trust is more likely to encourage treatment-seeking behavior upon development of first COVID-19 symptoms. This evidence underscores the importance of trust in health professionals as an element that influences vaccine uptake, and also speaks to the ability to follow COVID-19 preventative measures such as mask-wearing, handwashing, and physical distancing.

### Comparison With Prior Work

Lack of trust in religious organizations and leaders was associated with a decreased odds of vaccine hesitancy, while the opposite can also be concluded (ie, trust in religious organizations may be associated with increased vaccine hesitancy). Multiple surveys and polls have shown that certain faith communities within the United States are among the least vaccinated demographic groups, and contain the highest proportion of vaccine-hesitant individuals due to personal religious beliefs, wariness of science, and distrust in institutions [45-47]. This supports our finding that trust in religious organizations may be associated with increased vaccine hesitancy. Our finding also highlights the role religious leaders play in individuals’ decision-making regarding their health. If those who are vaccine-hesitant are more likely to trust their religious organizations for health and medical information, and alternatively, if in some cultures religious leaders are considered authorities on health and medical issues [48,49], religious organizations should be equipped with the tools and knowledge to provide constituents with evidence-based advice. Religious groups and leaders should not be excluded from conversations about COVID-19.

Access to health information and confidence in one’s ability to use health information may be one of the most critical aspects in decision-making regarding COVID-19 vaccine uptake. This study found that those who felt frustrated during their search for health information and those who lacked confidence in finding health information had greater odds of being vaccine-hesitant. Both of these factors are also related to inadequate health literacy, specifically eHealth literacy. Regular and reliable access to health information and services is associated with increased quality of life, and is known to prevent disease and disability due to early detection of illnesses and health conditions [50]. However, the literature also outlines the negative impact that health information found on the internet has on patient health. In one survey, 85% of physicians reported a patient bringing internet information regarding their health to a visit and expressed that it made the visits less efficient [51]. Additionally, information found on the internet regarding health and medicine can be harmful toward patient health since it can be misleading or entirely fabricated, whether intentionally or unintentionally [52,53]. For example, in the early stages of the pandemic, a video was widely shared on social media platforms that expressed cynicism and distrust in governmental figures and agencies, and made false claims about an eventual COVID-19 vaccine that had yet to be fully developed at the time [54]. This video changed the global and national conversation regarding the vaccines and the pandemic itself, causing significant damage to COVID-19 prevention efforts [55]. Misleading, false, and confusing health-related information can lead to frustration and deteriorate confidence when it comes to finding reliable information. This finding is echoed by the results in the Edelman Trust Barometer Report [12]. Studies have explored why conspiracy theories tend to take a stronghold on popular thought during traumatic large-scale events [56]; although there are several influential factors, one is a lack of access to accurate information due to the inherent novelty of the event [57,58]. The findings of this study underscore the importance of regaining trust in doctors and health professionals, and the importance of including nonhealth-related groups and organizations (ie, religious organizations) in the larger conversation about health education. The COVID-19 pandemic points to needs in partnerships for doctors and health professionals to work with trusted community lay health workers or community workers that may have greater trust in their community and reach to populations most at risk.

### Limitations

The results of this analysis should be considered in light of a few limitations. Primarily, all data collected were self-reported, which may be impacted by social desirability bias. However, the experiences reported were collected across a large sample. Further, the cross-sectional nature of this study limits our ability to assess temporality between our exposures (health communication components) and our outcome (vaccine hesitancy). Given the ongoing pandemic, the cross-sectional data do represent pandemic periods across variants. In the future, longitudinal data would be helpful to assess the impact of these critical components on increasing vaccine uptake. Finally, due to the convenience sampling method, the sample is not representative of the entire United States; therefore, the findings may not be generalizable to the national population. Specifically, 5.2% of our sample was vaccine-hesitant, which, in some states, is less than the proportion of the population who is vaccine-hesitant. According to the Centers for Disease Control and Prevention, the rate of vaccine hesitancy across states ranges from 2.9% to 27% [59]. In the United States, the vaccine-hesitant tend to be younger in age, less educated, and female, with some studies showing that non-Hispanic Black Americans have higher odds of vaccine hesitancy than non-Hispanic white Americans [60-62]. While our total sample consisted of primarily educated, non-Hispanic white, middle-aged women, our vaccine-hesitant sample tended to be less educated, younger, non-Hispanic white, and mostly women. While there are some similarities between our vaccine-hesitant sample and samples in the recent literature, there are also several differences, notably regarding race/ethnicity. Aside from demographic factors and related lived experiences contributing to vaccine hesitancy, others have found that political ideology is strongly associated with COVID-19 vaccination plans. Specifically, those who hold conservative ideologies tend to be more vaccine-hesitant [59-62]. Therefore, we conducted a posthoc exploratory analysis of political ideology and gender with vaccine-hesitant status, and found that significantly more vaccine-hesitant respondents identified as Republican (45% vs 12%, *P*<.001) and significantly more men identified as Republican (22% vs 13%, *P*<.001) in this sample. The political ideology divide could explain the slightly higher prevalence of men in the vaccine-hesitant sample within this study. The disparities between the vaccine-hesitant demographics in our sample compared with those of other studies is likely due to the primary recruitment strategy being through social media platforms among the study investigators (who are public health professionals). Despite this, these findings demonstrate the importance of trustworthiness of health professionals, adequate health literacy, and self-efficacy in decision-making to receive a COVID-19 vaccine. The small differences in the percentages found in our bivariate analysis could be due to the large and overpowered sample size.

### Conclusions

In 2019, the World Health Organization listed vaccine hesitancy as one of the top 10 threats to global public health [63]. While the factors explored in this analysis are critical elements in the discussion of vaccine hesitancy, there are a myriad of factors and external influences (eg, philosophical ideals, political affiliation, situational factors) that intersect with an individual’s decision-making regarding the COVID-19 vaccine, and vaccines in general [4]. The findings of this study underscore the importance of building trust between health professionals and their patients, involving nonhealth-related institutions in the conversation regarding health, and easing access to and raising self-efficacy in finding accurate and reliable health information.

## Abbreviations

AOR: adjusted odds ratio

## References

[1] COVID-19 Dashboard by the Center for Systems Science and Engineering (CSSE) at Johns Hopkins University (JHU).

[2] Honein MA, Christie A, Rose DA, Brooks JT, Meaney-Delman D, Cohn A, Sauber-Schatz EK, Walker A, McDonald LC, Liburd LC, Hall JE, Fry AM, Hall AJ, Gupta N, Kuhnert WL, Yoon PW, Gundlapalli AV, Beach MJ, Walke HT, CDC COVID-19 Response Team (2020). Summary of guidance for public health strategies to address high levels of community transmission of SARS-CoV-2 and related deaths, December 2020. MMWR Morb Mortal Wkly Rep.

[3] Meeting the challenges of vaccination hesitancy. Sabin Vaccine Institute.

[4] Getting to and sustaining the next normal: a roadmap for living with Covid. The Rockefeller Foundation.

[5] Coustasse A, Kimble C, Maxik K (2021). COVID-19 and vaccine hesitancy: a challenge the United States must overcome. J Ambul Care Manage.

[6] AJMC Staff (2021). A timeline of COVID-19 vaccine developments in 2021. The American Journal of Managed Care.

[7] Cassata C (2021). Doctors debunk 9 popular COVID-19 vaccine myths and conspiracy theories. Healthline.

[8] Jensen EA, Pfleger A, Herbig L, Wagoner B, Lorenz L, Watzlawik M (2021). What drives belief in vaccination conspiracy theories in Germany?. Front Commun.

[9] Love JS, Blumenberg A, Horowitz Z (2020). The parallel pandemic: medical misinformation and COVID-19 : Primum non nocere. J Gen Intern Med.

[10] Moran P (2020). Social media: a pandemic of misinformation. Am J Med.

[11] Bursztyn L, Rao A, Roth CP, Yanagizawa-Drott DH (2020). Misinformation during a pandemic. National Bureau of Economic Research.

[12] (2022). 2022 Edelman Trust Barometer. Edelman.

[13] Schiavo Renata (2013). Health communication: from theory to practice.

[14] Mheidly N, Fares J (2020). Leveraging media and health communication strategies to overcome the COVID-19 infodemic. J Public Health Policy.

[15] Chou WS, Budenz A (2020). Considering emotion in COVID-19 vaccine communication: addressing vaccine hesitancy and fostering vaccine confidence. Health Commun.

[16] Hocevar KP, Metzger M, Flanagin AJ, Parrott RL (2017). Source credibility, expertise, and trust in health and risk messaging. Oxford Research Encyclopedia of Communication.

[17] Slater MD, Rouner D (2016). How message evaluation and source attributes may influence credibility assessment and belief change. Journalism Mass Commun Quart.

[18] Lumsdaine AA (1954). Communication and persuasion. Educ Technol Res Dev.

[19] Bateman LB, Hall AG, Anderson WA, Cherrington AL, Helova A, Judd S, Kimberly R, Oates GR, Osborne T, Ott C, Ryan M, Strong C, Fouad MN (2022). Exploring COVID-19 vaccine hesitancy among stakeholders in African American and Latinx communities in the Deep South through the lens of the health belief model. Am J Health Promot.

[20] Trent M, Seale H, Chughtai AA, Salmon D, MacIntyre CR (2022). Trust in government, intention to vaccinate and COVID-19 vaccine hesitancy: A comparative survey of five large cities in the United States, United Kingdom, and Australia. Vaccine.

[21] Wu Y, Shen F (2022). Exploring the impacts of media use and media trust on health behaviors during the COVID-19 pandemic in China. J Health Psychol.

[22] Ouedraogo N, Ouakouak ML (2018). Impacts of personal trust, communication, and affective commitment on change success. J Organ Chang Manag.

[23] Huddy S (2015). Vulnerability in the classroom: instructor's ability to build trust impacts the student's learning experience. Int J Educ Res.

[24] Sørensen K, Van den Broucke S, Fullam J, Doyle G, Pelikan J, Slonska Z, Brand H, (HLS-EU) Consortium Health Literacy Project European (2012). Health literacy and public health: a systematic review and integration of definitions and models. BMC Public Health.

[25] Kricorian K, Civen R, Equils O (2022). COVID-19 vaccine hesitancy: misinformation and perceptions of vaccine safety. Hum Vaccin Immunother.

[26] Zhang H, Li Y, Peng S, Jiang Y, Jin H, Zhang F (2022). The effect of health literacy on COVID-19 vaccine hesitancy among community population in China: The moderating role of stress. Vaccine.

[27] Turhan Z, Dilcen HY, Dolu İ (2021). The mediating role of health literacy on the relationship between health care system distrust and vaccine hesitancy during COVID-19 pandemic. Curr Psychol.

[28] Schunk DH, DiBenedetto MK, Elliot J (2021). Self-efficacy and human motivation. Advances in Motivation Science, vol. 8.

[29] Rosenstock IM, Strecher VJ, Becker MH (2016). Social learning theory and the Health Belief Model. Health Educ Quart.

[30] Ajzen I (2002). Perceived behavioral control, self-efficacy, locus of control, and the theory of planned behavior. J Appl Soc Psychol vol.

[31] Prochaska JO, Velicer WF (1997). The transtheoretical model of health behavior change. Am J Health Promot.

[32] Guidry JP, Laestadius LI, Vraga EK, Miller CA, Perrin PB, Burton CW, Ryan M, Fuemmeler BF, Carlyle KE (2021). Willingness to get the COVID-19 vaccine with and without emergency use authorization. Am J Infect Control.

[33] Kim GY, Shin T, Son Y, Choi J (2022). Psycho-behavioural factors influencing COVID-19 vaccine hesitancy among Korean adults: The moderating role of age. J Adv Nurs.

[34] McElfish PA, Willis DE, Shah SK, Bryant-Moore K, Rojo MO, Selig JP (2021). Sociodemographic determinants of COVID-19 vaccine hesitancy, fear of infection, and protection self-efficacy. J Prim Care Community Health.

[35] Nelson D, Kreps G, Hesse B, Croyle R, Willis G, Arora N, Rimer B, Viswanath KV, Weinstein N, Alden S (2004). The Health Information National Trends Survey (HINTS): development, design, and dissemination. J Health Commun.

[36] Kwon N, Kim K (2009). Who goes to a library for cancer information in the e-health era? A secondary data analysis of the Health Information National Trends Survey (HINTS). Libr Inf Sci Res.

[37] Pavić Ž, Šuljok A (2022). PLoS One.

[38] Federal Poverty Level (FPL). Healthcare.gov.

[39] Smedley BD, Stith AY, Nelson AR, Committee on Understanding and Eliminating Racial and Ethnic Disparities in Health Care (2003). Unequal treatment: confronting racial and ethnic disparities.

[40] Khullar D (2018). Do you trust the medical profession?. New York Times.

[41] Blendon RJ, Benson JM, Hero JO (2014). Public trust in physicians--U.S. medicine in international perspective. N Engl J Med.

[42] Greene J, Ramos C (2021). A mixed methods examination of health care provider behaviors that build patients' trust. Patient Educ Couns.

[43] Thom DH, Hall MA, Pawlson LG (2004). Measuring patients' trust in physicians when assessing quality of care. Health Aff.

[44] Antinyan A, Bassetti T, Corazzini L, Pavesi F (2021). Trust in the health system and COVID-19 treatment. Front Psychol.

[45] Pulkkinen L (2021). White evangelical churches and the crisis of vaccine hesitancy. U.S. News.

[46] Dias E, Graham R (2021). White evangelical resistance is obstacle in vaccination effort. New York Times.

[47] Funk C, Tyson A (2021). Growing share of Americans say they plan to get a COVID-19 vaccine – or already have. Pew Research Center.

[48] Keshet Y, Popper-Giveon A (2021). "I took the trouble to make inquiries, so I refuse to accept your instructions": religious authority and vaccine hesitancy among ultra-orthodox Jewish mothers in Israel. J Relig Health.

[49] Kasstan B (2021). Vaccines and vitriol: an anthropological commentary on vaccine hesitancy, decision-making and interventionism among religious minorities. Anthropol Med.

[50] Access to health services. HealthyPeople.gov.

[51] Murray E, Lo B, Pollack L, Donelan K, Catania J, White M, Zapert K, Turner R (2003). The impact of health information on the internet on the physician-patient relationship: patient perceptions. Arch Intern Med.

[52] Wilson SL, Wiysonge C (2020). Social media and vaccine hesitancy. BMJ Glob Health.

[53] Islam MS, Sarkar T, Khan SH, Mostofa Kamal AH, Hasan SMM, Kabir A, Yeasmin D, Islam MA, Amin Chowdhury KI, Anwar KS, Chughtai AA, Seale H (2020). COVID-19-related infodemic and its impact on public health: a global social media analysis. Am J Trop Med Hyg.

[54] Kearney MD, Chiang SC, Massey PM (2020). The Twitter origins and evolution of the COVID-19 “plandemic” conspiracy theory. HKS Misinfo Review.

[55] Eberl J, Huber RA, Greussing E (2021). From populism to the “plandemic”: why populists believe in COVID-19 conspiracies. J Elect Public Opin Parties.

[56] Douglas KM, Uscinski JE, Sutton RM, Cichocka A, Nefes T, Ang CS, Deravi F (2019). Understanding conspiracy theories. Polit Psychol.

[57] Lor P, Wiles B, Britz J (2021). Re-thinking information ethics: truth, conspiracy theories, and librarians in the COVID-19 era. Libri.

[58] De Coninck D, Frissen T, Matthijs K, d'Haenens L, Lits G, Champagne-Poirier O, Carignan M, David MD, Pignard-Cheynel N, Salerno S, Généreux M (2021). Beliefs in conspiracy theories and misinformation about COVID-19: comparative perspectives on the role of anxiety, depression and exposure to and trust in information sources. Front Psychol.

[59] Kates J, Tolbert J, Rouw A (2022). The red/blue divide in COVID-19 vaccination rates continues: an update. Kaiser Family Foundation.

[60] Albrecht D (2022). Vaccination, politics and COVID-19 impacts. BMC Public Health.

[61] Sehgal NJ, Yue D, Pope E, Wang RH, Roby DH (2022). The association between COVID-19 mortality and the county-level partisan divide in The United States. Health Affairs.

[62] Ruiz JB, Bell RA (2021). Predictors of intention to vaccinate against COVID-19: results of a nationwide survey. Vaccine.

[63] Ten threats to global health in 2019. World Health Organization.

